# Promoting informed decision making about maternal pertussis vaccination: the systematic development of an online tailored decision aid and a centering-based group antenatal care intervention

**DOI:** 10.3389/fpubh.2024.1256337

**Published:** 2024-02-15

**Authors:** Charlotte Anraad, Pepijn van Empelen, Robert A. C. Ruiter, Marlies Rijnders, Katja van Groessen, Hilde M. van Keulen

**Affiliations:** ^1^Department of Work and Social Psychology, Faculty of Psychology and Neuroscience, Maastricht University, Maastricht, Netherlands; ^2^TNO Child Health, Netherlands Organization for Applied Scientific Research, Leiden, Netherlands; ^3^Stichting CenteringHealthcare Nederland, Zutphen, Netherlands

**Keywords:** intervention mapping, vaccination uptake, decision aid, informed decision making, maternal pertussis vaccination, centering pregnancy

## Abstract

**Introduction:**

Maintaining and enhancing vaccine confidence continues to be a challenge. Making an informed decision not only helps to avoid potential future regret but also reduces susceptibility to misinformation. There is an urgent need for interventions that facilitate informed decision-making about vaccines. This paper describes the systematic development of two interventions designed to promote informed decision making and indirectly, acceptance of maternal pertussis vaccination (MPV) in the Netherlands.

**Materials and methods:**

The 6-step Intervention Mapping (IM) protocol was used for the development of an online tailored decision aid and Centering Pregnancy-based Group Antenatal Care (CP) intervention. A needs assessment was done using empirical literature and conducting a survey and focus groups (1), intervention objectives were formulated at the behavior and determinants levels (2), theoretical methods of behavior change were selected and translated into practical applications (3), which were further developed into the two interventions using user-centered design (4). Finally, plans were developed for implementation (5), and evaluation (6) of the interventions.

**Results:**

The needs assessment showed that pregnant women often based their decision about MPV on information sourced online and conversations with their partners, obstetric care providers, and peers. Responding to these findings, we systematically developed two interactive, theory-based interventions. We created an online tailored decision aid, subjecting it to four iterations of testing among pregnant women, including those with low literacy levels. Participants evaluated prototypes of the intervention positively on relevance and usability. In addition, a CP intervention was developed with midwives.

**Conclusion:**

Using IM resulted in the creation of an online decision aid and CP intervention to promote informed decision making regarding MPV. This description of the systematic development of the interventions not only serves to illustrate design rationales, it will also aid the interpretation of the evaluation of the interventions, the development of future interventions promoting informed decision and acceptance of vaccines, and comparisons with other interventions.

## Introduction

With pertussis, commonly known as whooping cough, still prevalent in the Netherlands [36.8 cases per 100,000 in 2019 (1)], newborn infants who are not yet vaccinated are exposed to its health risks, potentially leading to hospitalization and, in rare cases, death (1, 2). To prevent pertussis in newborn infants, maternal pertussis vaccination (MPV) was introduced in the National Immunization Programme (NIP) in 2019 (3, 4). This vaccination is administered during pregnancy at 22 weeks of pregnancy, providing direct protection for infants immediately after birth until they can receive their first vaccination. In the Netherlands, pregnant women have the opportunity to receive MPV free of charge at or after 22 weeks of pregnancy at a youth health center, where the child vaccinations in the NIP are also administered. They receive an invitation letter and brochure about MPV from their obstetric caregiver (5). The current uptake of MPV in the Netherlands was estimated to be 70% in 2020 (1).

Currently, there are no studies done on the characteristics of those who accepted MPV versus those who did not accept MPV in the Netherlands. During the current project, MPV was introduced in the Netherlands. Prior to the introduction, our earlier study looked at determinants of the intention to accept MPV, giving us an idea of which factors are at play in the decision making process about MPV (6). This study included mothers and prospective parents with differing attitudes and intentions regarding MPV. Among others, beliefs about safety and effectiveness, moral and social norms, as well as anticipated regret were positively associated with vaccination intention. We will go into these determinants further in the results section.

In the Netherlands, in the decade prior to the COVID-19 pandemic, a decrease in vaccine uptake in the NIP over time (for example of recommended vaccinations for children), and a lower uptake of newly introduced vaccines in the NIP than expected were observed (1). Making informed decisions ensures that patients’ choice align with their values, helps prevent future feelings of regret, and reduces susceptibility to misinformation (7, 8). This can potentially result in higher vaccination rates, given that level of knowledge about a vaccine is often associated with a higher level of uptake of that vaccine (9). Given that MPV is a relatively new vaccination in the NIP, making it likely that people have questions about it, and the uptake is estimated to be 70% at the start of this study, we argue that it would be beneficial to promote informed decision making about MPV. In addition, for first-time parents, the MPV is the first vaccination decision in a series of vaccination decisions for their future child, making it especially relevant to ensure a positive experience (10).

This paper describes the systematic development of two interventions aimed at promoting informed decision-making about and uptake of MPV in the Netherlands. We used the Intervention Mapping (IM) framework to describe each step of the interventions’ development (11). IM provides a framework for using theory and empirical evidence to systematically develop behavior change interventions from a problem-based and participatory perspective. Interventions grounded in strong theoretical and empirical foundations tend to be more effective (12, 13). We advocate for transparent descriptions of health promotion interventions and their designs so that health promoters can replicate studies, and identify the conditions under which an intervention was (in)effective (14). Therefore, this paper describes our decision-making process and rationale at each step of the IM development process of the two interventions.

## Materials and methods

IM is an approach designed for the systematic development of health promotion and behavior change programs. It offers a framework that facilitates the design, planning, implementation, and evaluation of health promotion interventions.

IM consists of six steps. Step 1 entails constructing a logic model of the problem. In this step, we identify the behavioral and environmental causes of the problem, and the underlying determinants reflected as cognitions, beliefs, and feelings of members of the at-risk population and environmental decision-makers. To accomplish this, we reviewed literature, conducted a survey study [*n* = 611, described at (6, 15)] and a focus-group study. We conducted four focus-group interviews involving a total of 19 pregnant women who were aged 25–37, and recruited at midwife clinics. In the Netherlands, midwifery practices are the standard care option for prenatal care. In case of a complicated pregnancy, pregnant individuals go to a gynecologist instead. As 90% of pregnant individuals start their prenatal care at a midwifery clinic (16), recruitment at a midwifery clinic includes a wide range of members of the target group. Among the 19 participants, four already had a child. Nine were college or university-level educated, ten had a vocational or practical education. Four had already received the vaccination, and seven already had the intention to get the vaccination prior to the interview. The 1.5-h focus-groups were semi-structured and focused on factors associated with the decision to accept or refuse MPV, how pregnant individuals perceive the decision-making process, and their evaluation of sample information about MPV. The focus-groups were transcribed and analyzed using thematic coding.

In Step 2 of the IM protocol, performance objectives (POs) are formulated. These POs represent the (sub)behaviors that must be performed by the target group in order to reach the intervention goal. Also, for each PO and its determinants, change objectives are formulated. This results in a matrix outlining pathways for change in informed decision making and acceptance of MPV, serving as the core rationale for the intervention design. Step 3 concerns the design of the intervention program and its themes, components, scope, and sequence. This step includes the selection of theory-based intervention methods and the translation of these methods into practical applications, taking into account the parameters for the effectiveness of these methods. In Step 4, the methods and practical applications are being creatively translated into a cohesive intervention during the production phase, including pretesting of prototypes. In Step 5, the use of the intervention in real-life settings is carefully planned to ensure that the intervention will be adopted by the intended users and implemented according to the protocol to ensure sustained, long-term use of the intervention. The work done to ensure implementation does not take place after the development of the interventions, but takes place in parallel with the other steps. Finally, Step 6 concerns the planning of the process and effect evaluation of the intervention to measure program implementation and outcomes (11). Although the steps are presented as a linear process and outcomes of earlier steps inform later ones, it is important to note that IM is completed in an iterative way.

## Results

### IM step 1—needs assessment/logic model of the problem

#### Aims of the needs assessment

The needs assessment aimed to identify factors associated with the intention to accept MPV, and questions and information needs of pregnant individuals. This was examined with a qualitative study, conducting focus-group interviews with pregnant women (*n* = 19), a survey study (*n* = 611), and by reviewing literature (cited below).

#### Factors associated with the intention to accept MPV

The participants of the focus-group study indicated that reasons for accepting MPV included protection of their child, vaccine safety in the short and long term, recommendation from their GP or obstetric care provider, and the child being able to skip their first vaccination at 6 weeks of age. Additionally, they indicated that support from their partners and experiences from other women in their circles were important for their decision; some experienced social pressure when someone important to them opposed their decision. Conversely, reasons for refusing MPV were doubt, religious beliefs, a lack of trust in the NIP and feeling overwhelmed with the high amount of preventive or care interventions during pregnancy. These results were also found in the Dutch context in our previous survey study, where we studied determinants of the intention to accept MPV within the framework of the Theory of Planned Behavior and the Health Belief Model (6, 15, 17). Beliefs that the vaccine might cause harm was associated with a low vaccination intention, while beliefs that the vaccine was effective, safe, and beneficial for both mother and child were factors associated with a higher vaccination intention and uptake. Additionally, perceived susceptibility to infection and perceived severity of infection were related to a higher vaccination intention and uptake. Social norms, anticipated regret of accepting the vaccine, fear of the vaccine and of whooping cough, and decisional certainty were found to influence MPV intention. Under low levels of decisional certainty, intention to accept MPV was low, indicating that an ambivalent attitude about the vaccine leads to a lower uptake. Instead, promoting a robust, informed decision is likely to lead to a higher uptake of MPV (6). Our results are in line with findings from a systematic review by Kilich et al. (18). From this review, recommendation from a health-care professional to get vaccinated was also found to be of importance (13). Additionally, knowledge is considered a prerequisite for making an informed decision (8), and perceived control is thought to be of influence based on the theory of planned behavior (19). Finally, affect, in addition to cognitive factors, is thought to be of influence on vaccine-decision making (20). The factors listed about are also covered in models that specifically describe vaccine hesitancy. For example in the 3C model of vaccine hesitancy by the SAGE working group on vaccine hesitancy, in which determinants are categorized into confidence, complacency, and constraints (21).

#### Information needs

Participants in our focus-group study frequently sought online information about pregnancy and health, primarily on websites and social media. Another important source of information was other (previously) pregnant women. When presented with examples of information about MPV targeted at pregnant women, participants positively evaluated materials with a clean layout, a moderate amount of text with clear sub-headings, a reliable source, personal experiences of other women, and relevant images and explanatory videos. Conversely, information that was perceived as patronizing or condescending was evaluated negatively.

Among pregnant participants in our survey study (*n* = 202) (15), 55% a desire for assistance in making a decision about MPV (15). Of this group, 60% preferred a conversation with a healthcare professional, and 42% wanted to use an online decision aid. Most participants preferred to be informed by their obstetric caregiver. Information was desired about risks of side-effects in the mother and the baby, of the baby getting whooping cough, about the effectiveness of the vaccine, the symptoms of whooping cough, and possible alternatives for the vaccine. Information was preferably received through a brochure or letter (70%) or a website (49%).

### IM step 2—program outcomes and objectives—logic model of change

#### Program outcomes and objectives

Building on the identified problem and needs we formulated the following primary program outcome: pregnant women make an informed decision about MPV-uptake, and act upon that decision. The associated behavioral outcome is as follows: pregnant women make an informed decision about MPV-uptake and experience low or no decisional conflict. To achieve these outcomes, we have formulated the following performance objectives: (PO1) the pregnant women make an informed decision about the MPV, (PO2) make an appointment to get MPV, (PO3) ask questions about MPV if one has any, and (PO4) go to the Youth Health Centre to get MPV.

#### Behavioral determinants

Next, we identified the behavioral determinants that could potentially mediate a change in the specified performance objective, based on a review of existing literature (18) and our survey study (6). We selected all determinants deemed important based on the needs assessment and then selected those that were changeable. For example, for PO1 (making an informed decision), the selected determinants are: knowledge, attitude toward MPV, beliefs about safety, decisional certainty, injunctive norm, anticipated regret of vaccinating, beliefs about the effectiveness of MPV, negative and positive outcome expectancies of accepting MPV, social pressure, perceived control, positive and negative affect, risk perceptions and trust in the (provider of the) NIP (6, 18). For PO2, regarding making an appointment to get MPV, the selected determinants are knowledge, attitude, and perceived control about making the appointment. For PO3, regarding asking questions about MPV, the selected determinants are perceived control and trust in the (provider of) the NIP. For PO4, going to the Youth Health Centre to get the vaccine, selected determinants were knowledge, attitude, and perceived control. Supplementary Table S1 shows a complete overview of the performance objectives and the determinants targeted.

#### Change objectives

Change Objectives (COs) were subsequently formulated based on the intersecting of determinants with the performance objectives. Change objectives specify what the target audience should learn in relation to a determinant to fulfill the performance objective. Table 1 shows a sample of change objectives (for a complete overview, see Supplementary Table S1).

**Table 1:** Examples of change objectives, grouped per determinant.

Performance objectives	Determinants				
Pregnant women…	Knowledge	Attitude	Decisional certainty	Risk perceptions	Perceived control
PO1. Make an informed decision about the MPV	Recognize that MPV serves the purpose to protect her child once it’s born for several months until it can be vaccinated itself	Evaluate MPV positively. They recognize the health benefits of MPV for themselves and their unborn child	Feel on balance positively about the decision	Acknowledge the risk of side-effects of MPV, such as a painful arm, a red injection spot, body ache, fatigue or fever	Describe feeling in control of processing information about MPV
PO2. make an appointment to get MPV		Evaluate making the appointment as smoothly and positively			Describe feeling in control of making an appointment at the JGZ

A complete overview can be found in Supplementary Table S1.

### IM step 3—program design

This step describes the rationale of the intervention types chosen, based on the needs assessment and proximal program outcomes (change objectives). The selection of theoretical methods and their applications is based on the identified determinants and change objectives.

Because in the needs assessment pregnant women indicated searching for information online, and 42% in the survey indicated wanting to use an online decision aid, we decided to develop an online, tailored decision aid. Online interventions have the potential to reach large audiences at a low cost. Online tailoring is “a combination of strategies and information intended to reach one specific person based on characteristics that are unique to that person, related to the outcome of interest, and derived from an individual assessment” (22). Online tailored interventions have demonstrated great effectiveness to change health behavior than generic interventions (23).

However, even though online interventions can be effective, the reach of at-risk populations (i.e., those with low (health) literacy and socio-economic status) is more challenging (23, 24). Therefore, aside from making the online intervention as easily accessible as possible, we additionally developed an intervention based on the Centering methodology, a method that has become more common in the context of pregnancy (Centering Pregnancy; CP). CP is group-based prenatal care where individual consultations are replaced with group sessions, led by a midwife or other obstetric-care provider (25). Additionally, healthcare professionals play a potentially pivotal role in the decision making about vaccinations, and therefore an intervention where they are closely involved may have the potential to be effective (13). Because the group sessions are much longer (90–120 min) compared to individual sessions, there is more time for education, self-management, skills building, and building trust between caregiver and clients (26–28).

CP is associated with better pregnancy outcomes and an increase the initiation of breastfeeding compared to individual care. Pregnant women felt more empowered to voice opinions about care and indicated that they were more likely to feel that their wishes were listened to by care providers (29). Currently, CP has been adopted in approximately 35% of midwifery clinics in the Netherlands and has proven to be an effective strategy for reaching at-risk populations (29–31).

#### Theoretical methods and practical applications

For each determinant, we identified theory-based methods of change with the help of the taxonomy of behavior change methods of Kok et al. (32).

Knowledge and outcome expectancies were targeted using consciousness-raising (17, 33) about the MPV and pertussis in babies. Active learning (34, 35), feedback (36), and belief selection (19) were used to enhance the processing of information by participants. They answered questions about their beliefs before being given tailored feedback. Chunking (37) was applied to avoid information overload. In the CP intervention facilitative discussion were applied, encouraging participants to deliberate on the information, and encourage active participation, asking questions, in order to get to the issues that were most relevant for participants. Within CP, questions and concerns of pregnant women are leading for the conversation. Learning from other pregnant women by sharing and discussing experiences and considerations is encouraged (25).

To target attitude, we applied “feedback on benefits and barriers” (38) to help participants draw up a balance of their considerations. Furthermore in CP, arguments for and against MPV were discussed. For example, this was done by letting participants formulate questions and facilitating the group to find the answers.

Risk perception was targeted using scenario-based risk information (39). Risk information was presented using natural frequencies (e.g., 1 out of 100) to enhance the understandability of probabilities (40).

To target perceived control, injunctive norm, and social pressure, we used the methods “resistance to social pressure” (19) and “information about others approval” (41, 42). Participants were facilitated to prepare conversations and questions about MPV for important others or healthcare providers. Furthermore, we used modeling (36, 43), allowing participants to read about or talk about other’s experiences about how to deal with making the decision.

Details about how the methods and applications were used in the interventions are described in IM step 4. Supplementary Table S2 specifies which methods were used in each component of both interventions. Supplementary Table S3 provides a comprehensive overview of the theories selected for each determinant and their practical applications.

### IM step 4—program production

#### Theme, components, scope, and sequence

The online tailored decision aid and CP intervention were created in parallel. Both interventions can be used separately or combined. This section outlines the operationalization of the methods in both interventions.

#### The online decision aid

The online decision aid was created mobile-first in the form of a progressive web app because participants in the qualitative study (IM step 1) indicated a preference for using their mobile telephones most to search for pregnancy-related information online. During the development process, we aimed to meet the International Patient Decision Aid Standards (IPDAS) criteria for decision aids (44).

**Figure 1:**
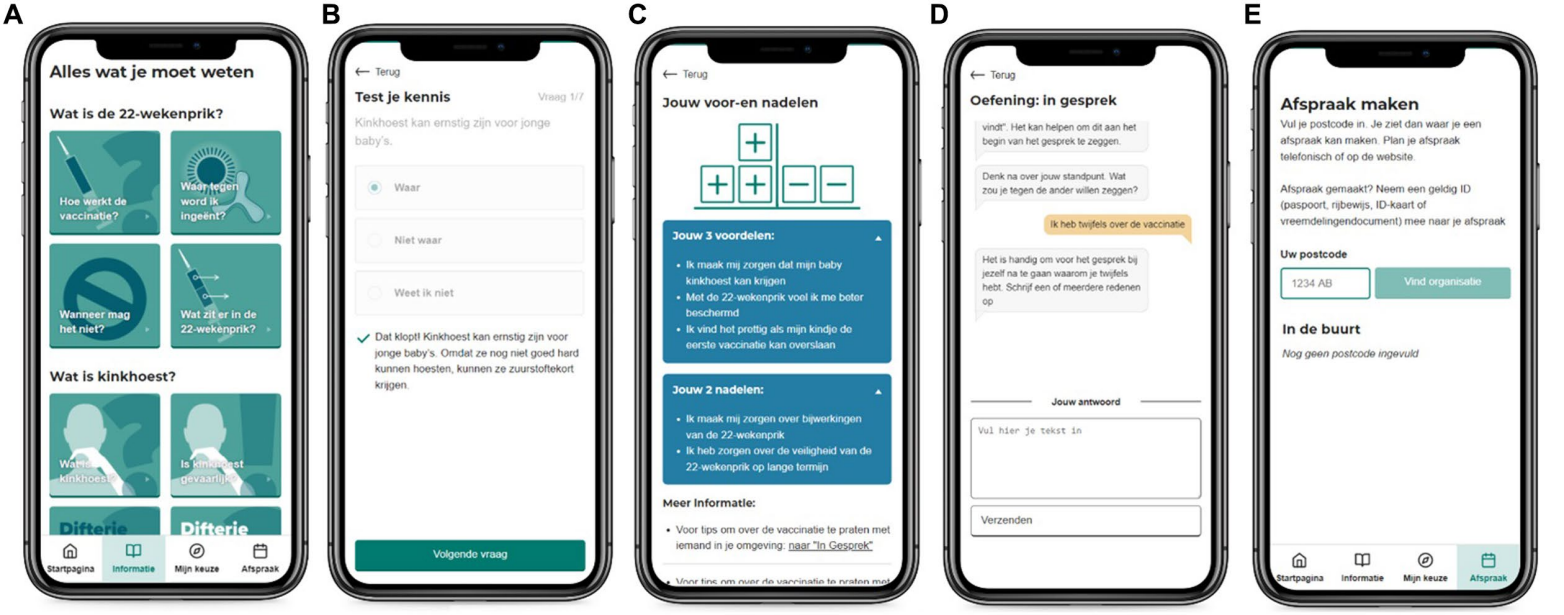
Screenshots of a selection of the website components: an overview of the information topics **(A)**, an example of a “test your knowledge” question and tailored feedback **(B)**, an overview page of the decisional balance with tailored pros and cons **(C)**, the “prepare a conversation” exercise **(D)**, the “make an appointment” page **(E)**.

The online decision aid consisted of three main components: (1) information tiles, (2) a module called “my choice,” and (3) a “make an appointment” module. Figure 1 presents screenshots of selected pages in the different components of the intervention. Participants were directed or “tunneled” from one page to the next, encouraging them to explore more components. They could also use the menu for navigation (45). Participants could visit the intervention as many times as they wished.

Participants were led, if they chose to follow the offered sequence, to the information component first (see Figure 1A). Because information is evaluated as more comprehensive when offered in the preferred mode (46), participants could choose if they preferred to watch a video, read text, or have the text read aloud. The text was chunked into basic information, automatically displayed, and “more information,” to prevent information overload (37). Sources of information were also provided, in line with the International Patient Decision Aid Standards (47). Videos showed a dialog between a pregnant woman and a midwife, along with visual organizers to explain concepts such as “how does the vaccine work?” and the rationale for administering MPV during pregnancy. The information pages stimulated active learning by providing “test your knowledge” questions. Participants could answer questions with true or false, and immediate feedback was provided (34–36, 48). The information provided on the information pages was checked for quality by a medical advisor of the National Institute for Public Health and the Environment, the RIVM.

The “my choice” component was divided into three submodules. The first, “test your knowledge” (Figure 1B), uses active learning and feedback to provide the most basic and relevant information about the vaccine (34–36, 48). The second, “weighing pros and cons” (Figure 1C), was aimed at improving decisional certainty by providing a tailored overview of the participants’ considerations about the MPV using a decisional balance (49). This exercise allows listing potential worries and feelings about MPV. Participants were presented with possible pros and cons of MPV and could indicate the extent to which these applied to them. Subsequently, participants were shown a customized overview of their results, without imposing a final judgment or recommendation. We did not include such a recommendation because some focus-group participants (IM step 1) negatively assessed materials that pressured them or directed them toward a specific choice. The third exercise was called “prepare a conversation about the vaccine” (Figure 1D). In this chat-like conversational module participants prepared for a conversation with a significant other, indicating what they wanted to gain from a conversation with an important social referent or health care provider, and what their feelings, needs, and questions were with regard to MPV. The module targeted dealing with social pressure and injunctive norm with regard to MPV by applying resistance to social pressure (19) and using non-violent communication (50). Participants received a customized overview of their responses that could be used in a conversation with an important other or healthcare provider.

The third component of the intervention was the “make an appointment” feature (Figure 1E). We included this to simplify the process of scheduling an MPV appointment, aiming to lower barriers for those who had decided in favor of the vaccine. We provided a postcode-based location finder where participants could make an appointment.

The DA meets the six qualifying criteria as defined by the IPDASi v4.0 guidelines (44). Aside from qualifying criteria, the IPDAS guidelines also contain certification criteria, quality criteria and evaluation criteria. The DA complies with five out of six certification criteria (four additional criteria that are only applicable to DAs about screening tests are not relevant for our DA). The certification criterion that our DA does not comply with, is the inclusion of author information and credentials in the DA. Out of 23 quality criteria (excluding the criteria for screening test DAs), the DA meets 18. A criterion that was not met was “The patient DA (or associated documentation) describes the quality of the research evidence used.” We did not do this in order not to overwhelm participants with information, especially since we aimed to make the DA inclusive for low-literate users. We did include references to the research evidence used. Another unmet criterion was “The patient DA includes authors’/developers’ credentials or qualifications.” The DA did include organizational credentials, but not that of the authors themselves. In addition, the DA did not report readability levels, but was instead tested with low-literate users to ensure readability. We also included a read-aloud option to improve readability. Whether the DA meets the evaluation quality criteria is assessed in the evaluation study. All other criteria with regard to development (the inclusion of users and professionals in the development process), evidence, guidance, values, probabilities and (balance of) information were met (44).

#### The centering pregnancy MPV information and decision making module

Within CP sessions participants are gathered in a circle. There are 10 sessions in total. Each session has an overall plan, but emphases may differ based on the group’s needs. Because of the long sessions (90 min) and the opportunity to socialize, group cohesion takes shape in which women feel supported and safe. The leadership of the midwife is transparent and facilitative. Women are empowered by being involved in check-ups and self-care activities, so they learn to understand how their body is changing during pregnancy. These principles of CP are founded by the Midwifery Model of Care, and derived from social-cognitive theory, targeting social support and self-efficacy enhancement (51). During each session, issues are discussed in an interactive way.

Within existing CP groups, the possibility to get the MPV is discussed during the second CP meeting, around 16 or 20 weeks of pregnancy. First, the midwife identifies the group needs, by asking participants about what they already know and think of MPV.

Second, the midwife decides which CP method to apply in order to convey information about MPV. Examples of CP methods, incorporating active learning, included quizzes that required participants to determine the accuracy of statements about MPV, followed by immediate feedback. Another method involved participants formulating questions and encouraging group discussions, all in line with principles that centered on questioning, reflection, and autonomy. Participants were facilitated to arrive to their own answers, a process during which the midwife guides the conversation and summarizes learning points by asking questions and encouraging participants to draw conclusions, based on the facts that were provided. Depending on the input of the participants, specific topics were further explored. The consequences of vaccinating versus not vaccinating are discussed, incorrect beliefs about safety and effectiveness of MPV are deconstructed, and correct beliefs about safety and effectiveness are strengthened, confirmed, or if needed, introduced.

Third, upon having discussed some of the facts around MPV, participants are encouraged to actively think about what the information they received means for their decision about MPV, and share this with the group if they wish to. Participants are further encouraged to voice any potential concerns and considerations. A method used to do this is to collectively make a list of pros and cons of getting the MPV and to individually write down those that are evaluated as most personally relevant. Participants share their thoughts on the MPV, and learn how to address these through discussing the MPV and voicing their concerns and beliefs, and seeing other participants do this.

Fourth, participants who are still in doubt about MPV are encouraged to contemplate, express, and pursue what they need to make a decision that they felt comfortable with. This might involve seeking individual consultation with the doctor providing the vaccine, or a conversation with the partner or other important person. Participants are then provided with practical information about how to get the vaccine, to make it as easy as possible to get MPV if they chose to do so.

#### Pre-testing intervention prototypes

We used user-centered design to create the online decision aid, aiming to meet the needs and user preferences of the target group. We involved the target group in four iterations during the development process. In all pre-tests, we involved pregnant women of diverse ages and backgrounds. The aim of the pre-tests was to get participants’ feedback on the intervention’s clarity, relevance, usability, and overall structure.

In the initial pre-test, a focus group consisting of six pregnant women was presented with a static intervention prototype. The prototype featured a feedback system where participants were first to answer a question before receiving tailored feedback. However, participants expressed a preference for immediate access to the information without the initial question. They also preferred not having to indicate in which form they wanted to see information: video or text, but to have both options directly available. Participants further wanted to have more control over the information they received.

In the second iteration, five pregnant women individually used an interactive prototype of the intervention during think-aloud sessions. Participants generally evaluated the intervention positively and found it relevant. Based on their feedback, we made several improvements, including shortening and chunking the texts, refining sub-topic divisions, incorporating more sub-headings, and consulting a text-writer specialized in writing health-information texts suitable for both low-literate and high-literate users.

The third iteration featured a full, interactive version of the intervention, with six pregnant women individually using the intervention individually during a think-aloud session. Participants indicated wanting to have more explanatory and guiding text in the intervention. We incorporated this feedback and included an introduction video on the homepage that explains the purpose of the web app. Participants further indicated a preference for direct feedback during the knowledge quiz, which we implemented. To improve usability, alterations in wording and placement of buttons were made based on the evaluation of the participants.

The fourth iteration was a usability test with four low-literate users in individual think-aloud sessions. These participants were not pregnant. The aim of the test was to evaluate the usability of the intervention for low-literate users, whether the intervention was easy to understand and navigate for them, and whether the core message of the intervention was understood. Participants indicated that they were still interested to learn more about the vaccination, but were discouraged to pursue this on the web app because of the amount of text. Therefore, we included the option to have the text read aloud using Readspeaker^®^. Additionally, we made several adjustments to icons and images used based on participants’ feedback to increase understanding.

After the final iteration, the intervention was tested further by members of the project group on various devices to ensure usability.

The CP intervention was developed by and in collaboration with midwives with extensive experience in applying CP methods and discussing vaccination in CP groups. The training was piloted with midwives trained to deliver CP. This process was embedded in a training for midwives that is part of the implementation and is described under IM step 5.

### IM step 5—program implementation plan

Implementation of a program happens at the end of the development process. However, planning for the implementation happens throughout the entire development. This paragraph describes the steps we took to *plan* the implementation, and make sure that the inventions aligned with the needs of potential implementation partners.

To implement the CP intervention, midwives already practicing CP are trained to deliver the CP-MPV intervention. During a 3-h training in groups of 12 midwives, the following steps are taken. First, midwives are invited to complete a self-evaluation form, to foster awareness about their own opinions about the MPV. Second, to start a conversation about vaccinations, an “across the line” exercise is done, where everyone indicates for example whether they ever had doubts about getting a vaccine, followed by a short discussion. Third, midwives are invited to adapt an interactive CP method for the context of MPV. The aim is to educate pregnant women about the immune system and the MPV, where to find and how to judge information about vaccines, and how to make the decision about MPV. Fourth, executing this was practiced in the plenary group, with participating midwives assuming roles with varying perspectives on MPV. This helps to enhance awareness of the perspectives of participants in CP groups. Furthermore, creating a safe environment to discussing the MPV, sources of information for midwives, and logistical matters such as the timing of the session are discussed.

The training was tested with a group of midwives (*n* = 12), after which the training was made more interactive, and exercises were included where midwives could apply their preferred CP-method on MPV, and practice this. After the first full training, participating midwives were consulted for feedback, and small adjustments were made to the training information materials, and timing of the exercises. After each subsequent training, feedback from participating midwives was gathered and where needed, adjustments were made.

To further optimize the implementation of the interventions upon evaluation, we formed a linkage group with stakeholders at the start of the project. This group included representatives from the National Institute for Public Health and the Environment, RIVM (the provider of the National Immunization Program), the Royal Dutch Organisation of Midwives (KNOV), the organization training for Centering based CP (CenteringZorg) the overarching organization of direct providers of the MPV to pregnant women (Dutch Youth Health Centre, NCJ), Radboud University, physicians from preventive Youth Health Care responsible for administering child and maternal vaccinations, and the Netherlands Patients Federation. Representatives of these institutions and groups advised on the qualitative study in the needs assessment, theme and scope of the interventions during the development, the interactive elements, the practicability, usability, flexibility of the interventions, the planned effect-evaluation, and the implementation plans. They were consulted at every step of the process, and provided, e.g., suggestions for which information examples to test in the focus-groups, which topics to prioritize in the interventions, etc. During the needs-assessment, this was done with a group meeting. For the other steps in the development, individual meetings were held between each advisor and one of the authors (CA), during which work was presented and feedback was collected. Feedback from the advisors was then discussed within the author team and integrated in the intervention.

The RIVM will get full control and management over the online decision aid if it turns out to be effective. They have been involved in the development phase to ensure a successful implementation. The CP intervention is owned and managed by CenteringZorg, who are also a member of the project team. The CP intervention is in line with existing CP care, also to ensure a successful implementation.

### IM step 6—evaluation plan

We planned to test the interventions in a semi-randomized controlled trial in order to assess their effects on informed decision-making, determinants of MPV uptake, and to check whether they influenced MPV uptake. In addition, we aimed to assess participants’ subjective evaluations of the interventions. The outcomes of the trial will be published separately. The study has been approved by the TNO Institutional Review Board (2018-050). The trial registration is available at https://www.onderzoekmetmensen.nl/en/trial/25018. This trial registration describes the initial trial design.

We planned to use a semi-randomized design because participating midwifery clinics could not be randomly assigned to the CP or control condition, as CP care is only offered in a limited number of clinics. However, due to the COVID-19 pandemic and the associated social-distancing measured in place at the time of the data collection, CP group-care could not safely take place and could therefore not be included in the large-scale trial. We then used a randomized controlled design for the evaluation of the online decision aid. We recruited pregnant individuals in the Netherlands through midwifery clinics and social media. Baseline measurements were conducted via questionnaires upon enrolment in the study (before or at 16 weeks of pregnancy). The intervention group was granted access to the decision aid in addition to standard information between 16 and 20 weeks of pregnancy, while the control group received only standard information. At 20 to 22 weeks of pregnancy, a follow-up questionnaire was conducted, including measures of informed decision making, decisional certainty, and acceptance and usability of the intervention. Vaccination status was derived from Praeventis, the National Immunization Register. Data were analyzed using an intention-to-treat approach, using mixed regression models for longitudinal data and logistic regression for vaccination uptake data.

When it became possible to resume CP group-care, we conducted a small-scale study to evaluate the feasibility and acceptability of the CP intervention. We interviewed midwives and participants who were involved in a CP session about MPV, and additionally administered questionnaires offered to all participants who participated in the sessions about MPV.

## Discussion

In this article, we have provided a detailed description of the systematic development of two complementary interventions promoting informed decision making about MPV during pregnancy. We created an online tailored decision aid for MPV decision-making. This included the provision of information using tailored feedback to existing beliefs, weighing pros and cons about the MPV, and a module to prepare a conversation about the MPV. Additionally, a CP session was developed that can be implemented in existing CP care settings. We applied a user-centered, iterative design to meet the needs of the target group, and participants evaluated the intervention positively. Although the interventions are designed to complement each other, especially to ensure targeting all sub-groups of the population of pregnant women, the interventions can easily be used independently and are not reliant on each other.

Vaccination programs still have lower uptake among lower-educated compared to higher-educated people (52, 53), and many (online) health interventions do not sufficiently reach at-risk populations such as those with low socioeconomic status (SES) and low literacy (24, 54, 55). We aimed to make the interventions suitable for those with low (health) literacy by involving low-literate users in the development of the online tailored decision aid, and by using a CP approach that has proven to be suitable for these populations.

Midwives play an important role as facilitators in the CP intervention. Therefore, it is important to note that their personal attitudes toward vaccination may impact the potential effectiveness of the intervention. Although we are not aware of studies in the Netherlands on attitudes about vaccination among midwives, a 2018 review of global literature on the topic shows that the majority of midwives supports vaccinations (56). However, there is a spectrum of beliefs present among midwives. The training that we have developed may help to deconstruct incorrect beliefs, but midwives who are critical of vaccination may be less inclined to follow the training. It is important that this is taken into account in the evaluation of the study.

We used IM to systematically develop the interventions, offering insight in the underlying rationales, and behavioral theories that informed their design. The IM intervention blueprint described in this article provides insight into the theories used in the different intervention components, helping to interpret the results of our evaluation study, aiming to identify causal mechanisms that contribute to intervention effects. Furthermore, this blueprint provides the opportunity to compare the interventions to other interventions on a theoretical level, for example in reviews or replication of studies in different contexts (12, 57). IM is a time-consuming process. But the blueprint created for the interventions can also advise the development of similar interventions for other vaccines or behaviors.

## Limitations

The small sample sizes in this study do not serve to evaluate the effectiveness of the intervention. They merely served to improve the intervention during the development process. We aimed to include a diverse group of pregnant individuals in terms of educational and cultural background. However, it is difficult to comment on generalizability of such a small sample. The larger evaluation study that was described under IM step 6 will provide statistically more robust data on use, acceptance and effectiveness of the interventions.

During the development of the interventions, we chose to target informed decision making rather than vaccination uptake. In addition, we aimed to reduce barriers to vaccination uptake once a decision had been made, and had the indirect aim to enhance vaccination uptake. This could be interpreted as conflicting with the decisional autonomy that a DA should respect and facilitate. The user-tests enabled us to guard a suitable balance of two-sided information for informed decision making. User input helped us redress the balance when a prototype of the DA was too favorable toward one decisional outcome.

## Conclusion

We developed two interventions aiming to promote informed decision making and to decrease decisional conflict about MPV. These interventions were developed using the IM framework, incorporating behavioral change methods from various theories. This systematic approach to intervention development will aid the interpretation of the process and effect evaluations of the interventions.

## Data availability statement

The datasets presented in this article are not readily available because participants did not consent to their data being shared. Requests to access the datasets should be directed to charlotte.anraad@maastrichtuniversity.nl.

## Ethics statement

The studies involving humans were approved by TNO Institutional Review Board (Reference number 2018-01). The studies were conducted in accordance with the local legislation and institutional requirements. The participants provided their written informed consent to participate in this study.

## Author contributions

CA: Conceptualization, Data curation, Formal analysis, Investigation, Methodology, Project administration, Writing – original draft, Writing – review & editing. PvE: Conceptualization, Funding acquisition, Investigation, Methodology, Supervision, Writing – review & editing. RR: Conceptualization, Funding acquisition, Investigation, Methodology, Supervision, Writing – review & editing. MR: Investigation, Supervision, Writing – review & editing. KvG: Investigation, Validation, Writing – review & editing. HvK: Conceptualization, Formal analysis, Funding acquisition, Investigation, Methodology, Project administration, Supervision, Writing – review & editing.
