# Supplementary material for: Promoting informed decision making about maternal pertussis vaccination: the systematic development of an online tailored decision aid and a centering-based group antenatal care intervention
Source: Front Public Health. 2024 Feb 15;12:1256337. doi: 10.3389/fpubh.2024.1256337 (PMC10902124; doi:10.3389/fpubh.2024.1256337)
Supplement: Supplementary file 1 [file Table_1.DOCX]

Table 1 of the supplementary material. Change objectives for each performance objective, grouped per behavioural determinant.

| **Performance objective** | **Determinant** | | | | | | | |
| --- | --- | --- | --- | --- | --- | --- | --- | --- |
|  | **Knowledge** | | | | **Attitude** | | **Positive outcome expectancies** | |
| PO1 Make an informed decision about the maternal pertussis vaccination | Describe that whooping cough is an infectious disease caused by a bacterium.   Describe that the bacteria can be transmitted via droplets in the air, via coughing and sneezing, physical contact such as shaking hands or kissing.  Recognize that whooping cough is prevalent in the Netherlands.  Describe that newborn children in their first few months of life are especially vulnerable to serious effects of whooping cough.   Describe that there is a vaccination against whooping cough during pregnancy at 22 weeks gestational age to protect a baby against pertussis.  Recognize that MPV serves the purpose to protect a child once it is born for several months until it can be vaccinated itself.  Recognize that currently MPV is the most effective strategy to protect a baby for pertussis in their first months of life.  Recognize that the whooping cough vaccination is freely available during pregnancy. | | Recognize that the MPV is administered in the arm.  Describe that the vaccination is a combination vaccine (DKTP) and consists of 1 injection.   Describe that a child can skip their first vaccination in the NIP (at 6 weeks) if the mother gets vaccinated during pregnancy.  Explain which strategies increase and decrease the risk of pertussis infection, including MPV, avoiding crowded places and not letting coughing people near their newborn.  Explain which factors put one at risk of getting pertussis and possibly passing that on to a baby, such as working with children or living in an area with a low vaccination coverage.  Recognize that there is no evidence for a link between MPV and adverse pregnancy outcomes, pregnancy complications and side-effects in the baby once it is born.   Recognize that MPV cannot give the mother or the baby whooping cough.  Describe where one can get MPV and how to make an appointment. | | Evaluate MPV positively by recognizing the health benefits of MPV for the mother and unborn child.   Recognize that the benefits outweigh the disadvantages of MPV  Evaluate the communication with important others about MPV positively. | | Describe the potential positive outcomes of MPV.   Describe that MPV reduces the risk of whooping cough for a baby in their first months of life.  Describe that MPV reduces the severity of whooping cough in a baby once it is born.   Describe that the risk of getting whooping cough is reduced by MPV, and that the severity of getting whooping cough is reduced by MPV.  Differentiate between facts and stories regarding the positive outcomes of MPV. | |
| **Performance objective** | **Determinant** | | | | | | | |
|  | **Negative outcome expectancies** | **Perceived control** | | **Social pressure** | **Positive affect** | **Negative affect** | | **Beliefs** |
| PO1 Make an informed decision about the maternal pertussis vaccination | Describe the potential negative outcomes of MPV.   Rcognize that after getting MPV, one might experience side effects such as a painful arm, a red injection spot, body-ache, fatigue or fever, and that some people will experience headache, nausea, vomiting, diarrhea, stomach ache and arm swelling.  Recognize that no evidence exists of a relationship between MPV and side effects in the baby.  Differentiate between facts and stories regarding the negative outcomes of MPV. | Describe feeling in control of processing information about MPV.  Describe feeling in control of deciding to get MPV.  Describe feeling in control of talking to others about the decision of getting MPV.  Pregnant woman expresses confidence about talking to important others about MPV. | | Recognize feelings of social pressure to accept or refuse the vaccine.  Demonstrate the ability cope with feelings of social pressure to accept or refuse the vaccine. | Describe feeling supported concerning any concerns about MPV.   Describe feeling reassured about any concerns and beliefs about MPV.  Describe feeling confident to ask questions about MPV in case of doubt. | Describe that having doubts about MPV is normal.  Describe not feeling stressed about any potential doubt about MPV.  Describe having low or no feelings of fear about MPV and its possible side-effects.  In case of feelings of fear about the vaccine and possible side-effects, recognize this and describe strategies to cope with these feelings. | | Recognize that MPV has proven to be safe for mother and child.  Recognize that the vaccine has proven to be effective in reducing infections with and severity of pertussis in newborn children.  Recognize that MPV is the most effective mean to protect a newborn baby against whooping cough.  Explain that living healthily is not an effective prevention strategy against pertussis  Recognize that breastfeeding is particularly effective against pertussis if the mother has received MPV, but not if she has not.  Recognize that going through whooping cough is not beneficial for a baby’s mental or physical health. |

| **Performance objective** | **Determinant** | | | | |
| --- | --- | --- | --- | --- | --- |
|  | **Risk perceptions** | **Injunctive norm** | **Descriptive norm** | **Trust** | **Decisional certainty** |
| PO1 Make an informed decision about the maternal pertussis vaccination | Acknowledge the risk of whooping cough and the severity of whooping cough of children in their first months of life.  Acknowledge the risk of side-effects of MPV, such as a painful arm, a red injection spot, body-ache, fatigue or fever.   Describe that there is a smaller risk of headache, nausea, vomiting, diarrhea, stomach ache and arm swelling.  Describe that if one has ever in had an allergic reaction to a vaccine, they should inform their MPV provider. | Formulate an opinion about MPV  Describe the opinion of important others (e.g. partner) about MPV   Express resistance to contrasting opinions about MPV towards important others | Recognize that other pregnant women decide to receive MPV. | Express trust in the MPV to protect babies from whooping cough.  Express trust in scientific research on MPV and information based on this.  Express trust in healthcare (the JGZ and midwives and/or gynecologist and/or GP) with regards to MPV.  Recognize that the government has implemented MPV in the NIP to reduce the number and the severity of pertussis infections in newborn babies.  Recognize that MPV is implemented to reduce pertussis in babies and not to benefit the pharmaceutical industry. | Recognize that feeling uncertainty about vaccination is common.   Describe feeling sure about the decision about MPV.  Describe feeling on balance positively about the decision. |

| **Performance objective** | **Determinant** | | | |
| --- | --- | --- | --- | --- |
|  | **Knowledge** | **Attitude** | **Perceived control** | **Trust** |
| PO2 Make an appointment to get MPV | Describe how to make an appointment to get MPV.   Describe what to bring to the appointment.  Describe what to expect of the appointment. | Evaluate making the appointment to get MPV as smoothly and positively. | Describe feeling in control of making an appointment at the JGZ. |  |
| PO3 Ask questions about MPV if one has any |  |  | Express confidence about talking to a healthcare professional about MPV  Express confidence about formulating questions about MPV and possibly writing them down before going to the appointment with the healthcare professional. | Express confidence in the answers provided by the healthcare professional when she asks questions. |
| PO4 Go to the Youth Health Centre to get MPV | Describe where to go to get the MPV | Evaluate getting the vaccine at the JGZ positively. | Describe feeling in control of the process of getting the vaccine and going to the Youth Health Centre. |  |

Table 2 of the supplementary material. Overview of the interventions, their components, main targeted determinants, and behaviour change methods used in each component

| **Intervention** | |  | **Components** | Knowledge | Attitude about MPV | Outcome expectancies | Beliefs safety | Beliefs effectiveness | Risk perception | Trust | Perceived control | Descriptive norm | Injunctive norm | Social pressure | Emotions/affect | Decisional certainty | **Methods of behaviour change** |
| --- | --- | --- | --- | --- | --- | --- | --- | --- | --- | --- | --- | --- | --- | --- | --- | --- | --- |
| Online-tailored decision aid | Information | | How does the vaccination work? |  |  |  |  |  |  |  |  |  |  |  |  |  | Consciousness-raising, active learning, scenario-based risk information, feedback, chunking, belief selection |
|  |  |  | Against what am I being vaccinated? |  |  |  |  |  |  |  |  |  |  |  |  |  |  |
|  |  |  | What is in the vaccination? |  |  |  |  |  |  |  |  |  |  |  |  |  |  |
|  |  |  | What is whooping cough? |  |  |  |  |  |  |  |  |  |  |  |  |  |  |
|  |  |  | How serious is whooping cough? |  |  |  |  |  |  |  |  |  |  |  |  |  |  |
|  |  |  | What are diphtheria and tetanus? |  |  |  |  |  |  |  |  |  |  |  |  |  |  |
|  |  |  | Is the vaccination safe? |  |  |  |  |  |  |  |  |  |  |  |  |  |  |
|  |  |  | Practical information |  |  |  |  |  |  |  |  |  |  |  |  |  |  |
|  |  |  | What are other people’s experiences? |  |  |  |  |  |  |  |  |  |  |  |  |  | Modelling |
|  | My choice | | Test your knowledge |  |  |  |  |  |  |  |  |  |  |  |  |  | Active learning, feedback |
|  |  |  | Weighing the pros and cons |  |  |  |  |  |  |  |  |  |  |  |  |  | Feedback on benefits and barriers, self-affirmation |
|  |  |  | Practice a conversation about the MPV |  |  |  |  |  |  |  |  |  |  |  |  |  | Resistance to social pressure |
|  | Make an appointment | | Information about the appointment |  |  |  |  |  |  |  |  |  |  |  |  |  | Decreasing barriers |
|  |  |  | Postal code-based location finder: *n/a* |  |  |  |  |  |  |  |  |  |  |  |  |  |  |
| CP intervention | Identifying needs | | n/a |  |  |  |  |  |  |  |  |  |  |  |  |  |  |
|  | Information | | Interactive information sharing (see description for examples) |  |  |  |  |  |  |  |  |  |  |  |  |  | Active learning, consciousness-raising, discussion, belief selection, scenario-based risk information. |
|  |  |  | Questions and answers |  |  |  |  |  |  |  |  |  |  |  |  |  | Belief selection. |
|  | Deliberation/discussion | | Sharing and discussing considerations |  |  |  |  |  |  |  |  |  |  |  |  |  | Arguments, modelling, resistance to social pressure, self-affirmation, information about others’ approval |
|  |  |  | Drawing up pros and cons |  |  |  |  |  |  |  |  |  |  |  |  |  | Decisional balance |
|  | Planning | | Planning what is needed to make the decision |  |  |  |  |  |  |  |  |  |  |  |  |  | Planning coping responses |
|  |  |  | Planning what is needed to get the vaccine |  |  |  |  |  |  |  |  |  |  |  |  |  | Decreasing barriers |

Table 3 of the supplementary material. Behavioural change methods and their applications in the intervention, and the targeted determinants. All determinants were targeted in order to achieve performance objective 1, and additionally, perceived control was also used to achieve performance objective 2. Determinants are ordered from most targeted in the intervention to least targeted in the intervention.

|  | **Online decision aid** | | **CP intervention** | |
| --- | --- | --- | --- | --- |
| **Determinant** | **Methods** | **Strategy** | **Methods** | **Strategy** |
| Knowledge | Consciousness raising (Health Belief Model, Precaution-Adoption Process Model, Trans-Theoretical Model)  Active learning (Elaboration Likelihood Model, Social Cognitive Theory)  Feedback (Theories of Learning)  Chunking (Theories of Information processing) | Two-sided information was presented per subject, and in levels, with the possibility to click to reveal more information. Both videos and textual information was presented so participants could choose how to process information based on their preferences. Subjects were: What is the whooping cough vaccination during pregnancy? (i.e. How does the vaccination work? What is in the vaccination?) What is whooping cough? Is the vaccination safe? Are there side-effects? What do other think of the vaccination?  Per subject, a quiz-question was available, with direct feedback.  Participants could do a knowledge-quiz in the ‘my choice’ section, where feedback was also provided | Active learning (Elaboration Likelihood Model, Social Cognitive Theory)  Discussion (Elaboration Likelihood Model) | During the CP session, the midwife chose an interactive method to encourage active learning. An example of an interactive CP method is using an exercise where participants were asked to indicate if a question is true or false, after which they are given immediate feedback, or letting participants write down their questions and encouraging others to discuss the answers. Participants were facilitated to conclude answers to questions themselves, a process during which the midwife guides the conversation by asking questions and encouraging participants to conclude answers based on the facts that were provided. Which sub-topics were further explored depended on the input of the participants. |
| Attitude | Feedback on benefits and barriers (decisional balance) | Participants were provided with a decisional balance with pros and cons of MPV, based on their concerns and values. For example they were asked if they agreed with: ‘I am concerned that my baby might get whooping cough’ and ‘I would prefer my baby to be able to skip the first vaccination at two months of age’, resulting in a visual overview of personal pros and cons of MPV. Questions could also be skipped when found irrelevant. The content of the questions was based on focus-group interviews (see step 2) and was balanced between pros and cons.  Participants were provided with quotes from pregnant women about choosing about MPV. | Feedback on benefits and barriers  Arguments (Communication Persuasion-Matrix, Elaboration Likelihood Model) | Upon having discussed some of the facts around MPV, participants were encouraged to actively think about what that information meant for their decision about MPV. Participants were further encouraged to voice their concerns and considerations about MPV. The consequences of vaccinating versus not vaccinating were discussed, and arguments for and against the MPV could be shared. An example of a method that could be used by the midwife, is to collectively make a list of pros and cons of getting the MPV, and to individually write done those that are evaluated as most personally relevant. |
| Outcome expectancies | Consciousness raising (Health Belief Model, Precaution-Adoption Process Model, Trans-Theoretical Model)  Belief selection (Theory of Planned Behaviour)  Active learning (Elaboration Likelihood Model, Social Cognitive Theory)  Feedback (Theories of Learning) | Participants were shown two-sided information about MPV (see above). Per subject, a quiz-question was available, with direct feedback.  Participants could do a knowledge-quiz in the ‘choice’ section | Consciousness raising (Health Belief Model, Precaution-Adoption Process Model, Trans-Theoretical Model)  Belief selection (Theory of Planned Behaviour) | The consequences of vaccinating versus not vaccinating were discussed and explored. Incorrect beliefs about safety and effectiveness of MPV were weakened, and correct beliefs about safety and effectiveness were strengthened, confirmed, or if needed, introduced. |
| Perceived control | Resistance to social pressure (Theory of Planned Behaviour) | Participants could practice a conversation about MPV in an automated virtual chat, where they could fill out several questions to get an overview of what they want to say, what they need, and want to ask. They were provided with an overview of their answers.  We provided practical information about how to get the vaccine, and what to expect during the process of getting the vaccine, to remove barriers. | Self-affirmation (Self Affirmation Theory)  Modelling (Social-Cognitive Theory, Theories of Learning) | Participants were provided with practical information about how to get the vaccine. Further, through discussions in the group, participants learned from each other, and the way they made decisions about vaccinations. |
| Social pressure | Resistance to social pressure (Theory of Planned Behaviour) | In the information, it was emphasised that MPV is a choice and that accepting it is voluntary.  Participants could practice a conversation about MPV in an automated virtual chat, and they were provided with an overview of their answers. | Resistance to social pressure (Theory of Planned Behaviour)  Modelling (Social-Cognitive Theory, Theories of Learning) | Through discussions in the group, participants learned from each other, and the way they handle social pressure. |
| Emotions | Self-affirmation (Self Affirmation Theory) | It was emphasised that it is important that participants made an informed decision that they felt positive about. There was space in the decisional balance to become aware of worries and feelings about the MPV. By addressing concerns seriously in the information, yet debunking myths firmly, we aimed to make participants feel heard. | Self-affirmation (Self Affirmation Theory) | It was emphasised that it is important that participants made an informed decision that they felt positive about.  During the session, there was time and space to express emotions, worries and concerns surrounding the MPV. |
| Beliefs about safety  and beliefs about the effectiveness of MPV | Belief selection (Theory of Planned Behaviour) | Incorrect beliefs about safety and effectiveness of MPV were weakened, and correct beliefs about safety and effectiveness were strengthened, confirmed. This was done in the information and in the quiz-elements of the intervention. | Belief selection (Theory of Planned Behaviour) | Incorrect beliefs about safety and effectiveness of MPV were weakened, and correct beliefs about safety and effectiveness were strengthened, confirmed, or if needed, introduced. |
| Risk perception Pertussis | Scenario based risk information (Precaution-Adoption Process Model) | Risk information was presented using natural frequencies (e.g. 1 out of 100) to improve understandability of probabilities. | Scenario based risk information (Precaution-Adoption Process Model)  Personalise Risk (Precaution-Adoption Process Model) | Risk information was presented using natural frequencies (e.g. 1 out of 100) to improve understandability of probabilities. Personal risk factors of contracting whooping cough, such as being exposed to a lot of people, where discussed. |
| Injunctive Norm | Information about others approval (Theory of Planned Behaviour; Social Comparison Theory) | Participants could practice a conversation about MPV in an automated virtual chat, and they were provided with an overview of their answers. | Information about others approval (Theory of Planned Behaviour; Social Comparison Theory)  Modelling (Social-Cognitive Theory, Theories of Learning) | Participants during the session shared their thoughts on the MPV, and learned how to address these in other situations through discussing the MPV and voicing their concerns and beliefs, and seeing other participants do this as well. |
| Descriptive Norm | Information about others approval (Theory of Planned Behaviour; Social Comparison Theory) | Participants could read about which countries in the world already use MPV. | Information about others approval (Theory of Planned Behaviour; Social Comparison Theory) | Participants during the session shared their thoughts on MPV. |
| Trust in the NIP | n/a | The different parties involved in the MPV and their roles were explained in the information, as well as the rationale for the government to implement MPV in the National Immunisation Programme. | n/a | Background information about the NIP was provided, as well as the rationale for the government to implement MPV in the National Immunisation Programme. |
| Decisional certainty | Planning coping responses (Theories of Self-Regulation)  Decisional balance | Participants were offered suggestions of what to do when uncertain, such as speak to a professional, their partner, gather more information, or weigh the pros and cons. Furthermore it was emphasised that feeling uncertainty is normal. | Planning coping responses (Theories of Self-Regulation) | Participants who were still in doubt about MPV upon the session about MPV, were encouraged to think about, express and pursue what they needed to make a decision that they felt good about, for example an individual consultation with the Youth Doctor providing the vaccine, or a conversation with the partner. |
